# Evidence for a tubular basement membrane–cilia connection in autosomal dominant polycystic kidney disease pathogenesis

**DOI:** 10.1172/JCI210031

**Published:** 2026-09-15

**Authors:** Caroline R. Sussman, Peter C. Harris

**Affiliations:** 1 Division of Nephrology and Hypertension,; 2 Mayo Clinic Pirnie Translational Polycystic Kidney Disease Center, and; 3 Department of Biochemistry and Molecular Biology, Mayo Clinic, Rochester, Minnesota, USA.

## Abstract

Autosomal dominant polycystic kidney disease (ADPKD), mainly driven by pathogenic variants in *PKD1* and *PKD2*, is the most common inherited cause of kidney failure. Details of ADPKD pathogenesis are incompletely resolved, but primary cilia are an integral component. There is also evidence for changes to the tubular basement membrane (TBM) in early disease. In this issue of the *JCI*, Mazloum and colleagues link cilia-dependent, PKD1-mediated regulation of the TBM to ADPKD pathogenesis. Using in vivo, ex vivo, tubule-on-chip, and cellular models of ADPKD, they connect cilia-dependent tubule dilation and TBM thinning to early-stage cystogenesis. Moreover, they identify a cilia-dependent TBM remodeling expression signature in affected tubules and suggest that PC1 loss compromises TBM stiffness. By integrating roles for cilia at the apical membrane and extracellular matrix at the basolateral membrane in cystogenesis, this work highlights the TBM as an additional area for investigation of therapeutic and biomarker discovery in ADPKD.

Autosomal dominant polycystic kidney disease (ADPKD) is the most common inherited cause of kidney failure. Pathogenic variants in *PKD1* or *PKD2*, encoding the polycystins PC1 and PC2, account for most cases. These disease-associated gene variants promote abnormal renal tubule growth, driving formation and growth of cysts that structurally alter the kidney and diminish the function of normal renal tubules as the disease progresses. Tolvaptan, a vasopressin receptor 2 antagonist, is currently the only pharmacological therapy approved for ADPKD. Though this treatment can slow disease progression, most patients eventually progress to kidney failure, for which dialysis or kidney transplantation are the only remaining treatment options. Thus, identifying targetable pathogenic mechanisms in ADPKD is critical to advancing therapeutic options.

## Linking primary cilia and basement membrane to cystogenesis

The major proteins associated with ADPKD, PC1 and PC2, are thought to form a Ca^2+^ channel complex (1). The PC complex appears to localize at the primary cilium, a single signaling antennae found protruding from most cells, including the apical surface of the epithelial cells lining kidney tubules. Multiple lines of evidence implicate primary cilia in the pathogenesis of ADPKD and specifically cystogenesis. This evidence includes subcellular localization of the *C*. *elegans* *PKD1* and *PKD2* homologs, *lov-1* and *pkd-2* (2), and mammalian PC1/2 to cilia (3, 4). Further linking cilia to pathogenesis, monoallelic variants of *IFT140*, encoding a protein involved in ciliary intraflagellar transport (IFT) of proteins, are the third most common cause of ADPKD (5). Moreover, cilia are implicated in diseases, including autosomal recessive PKD (driven by mutations in cilia-localized fibrocystin), and ciliopathies, which often have a cystic/fibrotic kidney and liver phenotype (6).

The timing of PC1 or PC2 loss in conditional knockout (CKO) mouse models influences disease severity, with much more rapid cystogenesis occurring in models that lose PC1 or PC2 by postnatal day 13 than later time points (7). Inducing loss of renal cilia, such as with loss of the structural ciliary gene *Ift120* or *Kif3a*, also results in kidney cysts, and severity is temporally regulated to a degree similar to that in *Pkd1* or *Pkd2* CKO models, although phenotypes are milder than in those models (8). Paradoxically, inducing combined loss of PC1 or PC2 and cilia results in a much milder phenotype than PC loss alone (9). This has been explained by a signaling activity termed cilia-dependent cyst activation (CDCA) that promotes cyst growth but is normally suppressed by PC complex signaling. Various pathways have been implicated in PC complex signaling, including cAMP (a downstream target of tolvaptan), Ca^2+^, WNT, mTOR, Hippo, and PC1 cleavage pathways (10), while the transcriptional regulator GLIS2 has been suggested as a critical component of CDCA signaling (11).

To a lesser extent, the tubular basement membrane (TBM) has also been implicated in cystogenesis. The kidney TBM provides structural support for epithelial cells, contributes to semipermeable barrier properties, and has an important role in normal tissue development and repair. Traditionally, ADPKD has been associated with thickening and disorganization of the TBM (12), but this likely reflects the widespread fibrosis found in late-stage disease. However, studies in *Pkd1*-deficient ADPKD mouse models have implicated integrins in early cystogenesis, including observations that disrupting integrin-β1 dramatically improved cystogenesis and targeting activin slowed pathogenesis (13–15). In addition, combining *Pkd1*^CKO^ with deletion of *Adamts1*, which cleaves the ECM component versican in the TBM, reduced cyst growth and improved kidney function and survival compared with *Pkd1*^CKO^ alone (16). Further supporting the TBM’s role in cystogenesis, monoallelic pathogenic variants of TBM-localized collagens can cause occasional cysts (17). Other phenotypes present in ADPKD, including vascular fragility (evident as increased risk of aneurysm) and increased risk of abdominal wall hernias also suggest broader weakness of the ECM in ADPKD.

## Cilia-dependent TBM alterations in ADPKD pathogenesis

In this issue, Mazloum et al. provide evidence uniting ciliary and TBM pathogenic processes associated with cystogenesis (18). Their tour de force study involved CKOs of *Pkd1* and structural ciliary genes (*Ift120* or *Kif3a*); loss of peroxidasin (*Pxdn*), encoding a protein regulating TBM stiffness; various RNA-seq studies, including from dissected mouse tubule segments; and single tubule perfusion, tubule-on-chip, and unilateral ureteral obstruction (UUO) methods. In the *Pkd1*^CKO^ model of late-onset PKD, the first sign of cyst development was tubular distension. This was attributed to cellular stretching rather than cellular proliferation in the collecting duct (CD) and distal tubule. Coincident cilia loss blocked these changes. Mazloum et al. next showed that tubular distension was associated with TBM thinning and deposition of heparin sulfate proteoglycans, which are essential components of the ECM. The authors made the case that these changes were distinct from the cystogenesis associated with cilia loss alone in both late- and earlier-onset PKD models. Bulk RNA-seq from dissected CDs and proximal tubules (PTs) identified a TBM expression signature associated with PC1 loss in the CDs but not PTs. Again, coincident cilia loss abrogated this signature. A prior study of single-nucleus RNA-seq of human ADPKD tissue also pointed to a TBM remodeling expression signature, although this may reflect later-stage fibrosis in the patient population studied (19). Mazloum et al. next explored a possible role for GLIS2 in the pathway linking PC signaling and the TBM, showing that *Pkd1*^CKO^ *Glis2*^CKO^ mice had reduced tubule dilation and cellular stretching, but some TBM thinning remained.

Studies using the isolated tubule perfusion system provided further evidence of TBM abnormalities associated with PC1 deficiency (18). Increased luminal pressure induced by this system resulted in increased tubule diameter at low pressure in *Pkd1*-deficient CDs relative to *Pkd1*-deficient PTs and WT CDs, an effect that was cilia dependent. Tubule-on-chip studies using murine inner medullary CD cells showed greater dilatation in *Pkd1*^KO^ cells compared with WT when cells were cultured on ECM scaffolds with a lower collagen content, translating to a lower stiffness threshold. Along these lines, previous work has suggested that a *COL4A1* pathogenic variant exacerbated the PKD2 phenotype in one family (20). Mazloum et al. therefore used the *Pxdn*^KO^ mouse to induce a decrease in TBM stiffness. In this model, while decreased TBM stiffness alone did not induce cysts, tubular dilations were greater in combined *Pkd1*^CKO^ *Pxdn*^KO^ mice (especially in males) compared with *Pkd1* loss alone.

A final approach was to examine cystogenesis after UUO in *Pkd1*^CKO^ versus WT mice (18). Enhanced cystogenesis in the CD of *Pkd1*^CKO^ mice was cilia dependent and associated with cellular stretching. Following UUO resolution, neither the kidney weight/body weight ratio nor tubule dilation was reversed, indicating that cysts can be induced by a temporary obstruction. Obstruction has been previously suggested as a mechanism contributing to cyst growth in PKD animal models (21), and there is recent evidence that induced kidney crystal deposition can increase the severity of ADPKD (22) and that kidney stones are a risk factor for ADPKD progression (23). These studies are consistent with earlier ones indicating that renal injury can profoundly increase the severity of PKD in mouse models of ADPKD (24, 25). However, given the multiple consequences of UUO, including increased fibrosis, whether disease severity is wholly due to increased intraluminal pressure in the UUO model is unclear.

## Conclusions

**Figure 1:**
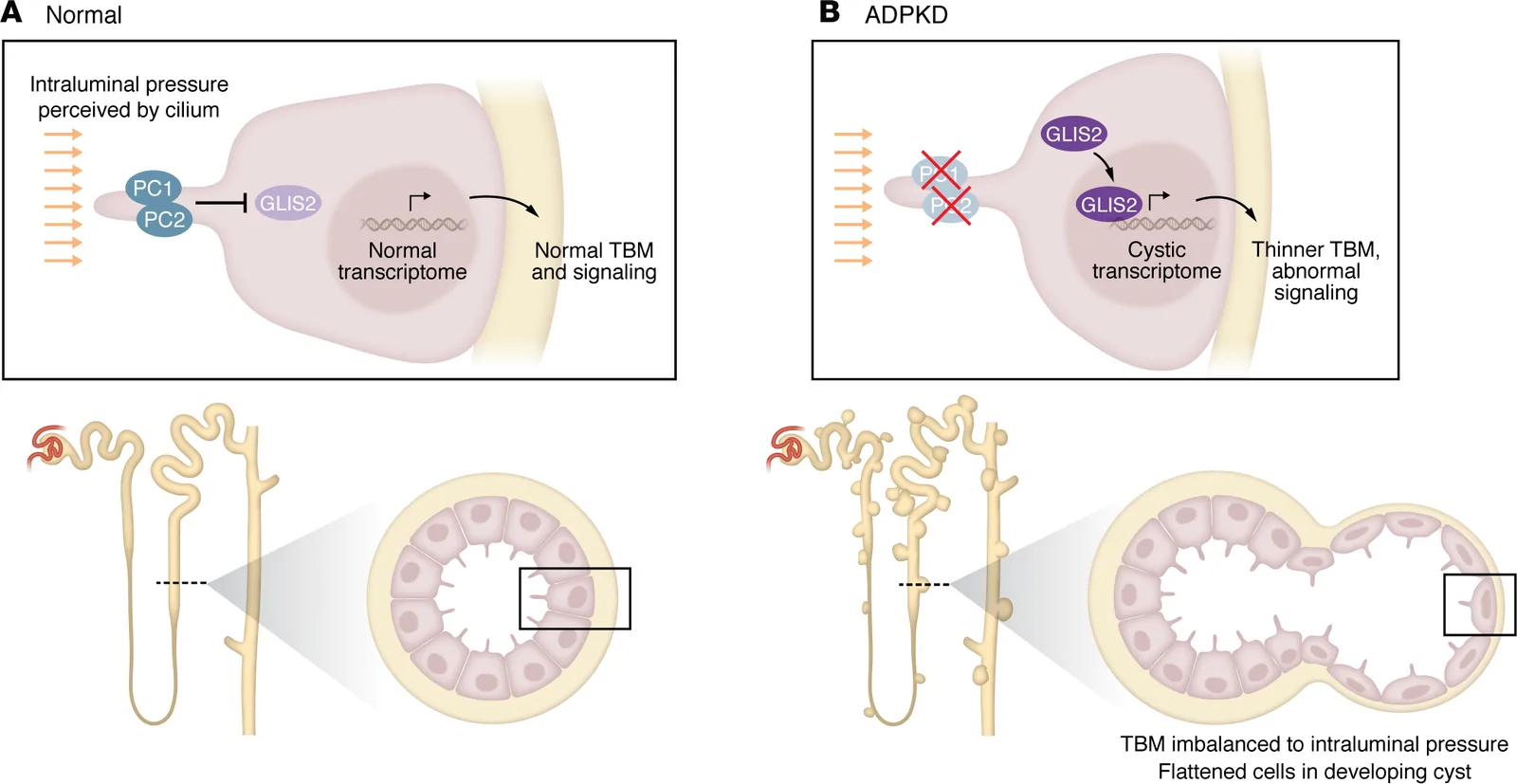
TBM changes associated with PC complex insufficiency are an early event in ADPKD cystogenesis. The findings of Mazloum et al. (18) support the proposed connection between primary cilia on the apical surface of a tubular epithelial cell, which contain the PC complex (comprising ADPKD-associated proteins PC1 and PC2), and the TBM adjacent to the basal membrane. (**A**) Normally the PC complex may detect intraluminal pressure and send a signal to the TBM to regulate its thickness and compliance. This may be mediated by the PC complex blocking the transcriptional regulator GLIS2, whose activity is associated with cystogenesis via the CDCA. (**B**) In ADPKD, loss or reduction of PC1 or PC2 below a critical threshold impairs PC complex signaling. This reduces the ciliary ability to detect/signal intraluminal pressure appropriately, leading to activation of the CDCA and resulting in thinning of the TBM and increased compliance plus cellular stretching, which Mazloum et al.’s study associated with cyst initiation.

Overall, Mazloum et al. (18) make a strong case for changes in the TBM associated with PC1 loss/reduction as an early event in cyst development, highlighting another mechanism to explore for therapeutic development in ADPKD (Figure 1). Compounds strengthening the TBM in early disease stages may be of value; however, given the association of TBM thickening and disorganization with the fibrosis characteristic of later-stage disease, timing and specificity of treatment might be important. The study also emphasizes that trying to minimize tubule blockage, for instance by reducing stone formation, may be of value. Of note, the sole current pharmacological treatment for ADPKD, tolvaptan, promotes high levels of water intake, which is also a recommendation for stone prevention. That TBM changes in ADPKD are dependent on cilia supports the central role of cilia in pathogenesis, as opposed to TBM changes being primarily an effect of the PC complex directly interacting with the TBM. While the components linking the PC complex, cilia, and the TBM are still largely unknown, GLIS2 is hinted at in this study. Elucidating these relationships and determining whether the ciliary PC complex has a mechanosensory function, such as measuring intralumenal pressure, are important areas for further study.

## Conflict of interest

PCH receives licensing fees for the Pkd1RC model.

## Funding support

This work is the result of NIH funding, in whole or in part, and is subject to the NIH Public Access Policy. Through acceptance of this federal funding, the NIH has been given a right to make the work publicly available in PubMed Central.

- National Institute of Diabetes and Digestive and Kidney Diseases grant DK058816.

## Version 1 09/15/2026

Electronic publication
